# Replication of LDL GWAs hits in PROSPER/PHASE as validation for future (pharmaco)genetic analyses

**DOI:** 10.1186/1471-2350-12-131

**Published:** 2011-10-06

**Authors:** Stella Trompet, Anton JM de Craen, Iris Postmus, Ian Ford, Naveed Sattar, Muriel Caslake, David J Stott, Brendan M Buckley, Frank Sacks, James J Devlin, P Eline Slagboom, Rudi GJ Westendorp, J Wouter Jukema

**Affiliations:** 1 Department of Cardiology, Leiden University Medical Center, Leiden, the Netherlands; 2 Department of Gerontology and Geriatrics, Leiden University Medical Center, Leiden, the Netherlands; 3 Netherlands Consortium of Healthy Ageing, Leiden, the Netherlands; 4 Robertson Center for Biostatistics, University of Glasgow, Glasgow UK; 5 BHF Glasgow Cardiovascular Research Centre, Faculty of Medicine, Glasgow, UK; 6 Vascular Biochemistry Section, Institute of Cardiovascular & Medical Sciences, University of Glasgow, Glasgow, UK; 7 Institute of Cardiovascular and Medical Sciences, School of Medicine, University of Glasgow, Glasgow, UK; 8 Department of Pharmacology and Therapeutics, University College Cork, Ireland; 9 Department of Nutrition, Harvard School of Public Health, Boston, Massachusetts, USA; 10 Celera, Alameda, California, USA; 11 Department of Molecular Epidemiology, Leiden University Medical Center, Leiden, The Netherlands; 12 Durrer Center for Cardiogenetic Research, Amsterdam, The Netherlands; 13 Interuniversity Cardiology Institute of the Netherlands, Utrecht, The Netherlands

## Abstract

**Background:**

The PHArmacogenetic study of Statins in the Elderly at risk (PHASE) is a genome wide association study in the PROspective Study of Pravastatin in the Elderly at risk for vascular disease (PROSPER) that investigates the genetic variation responsible for the individual variation in drug response to pravastatin. Statins lower LDL-cholesterol in general by 30%, however not in all subjects. Moreover, clinical response is highly variable and adverse effects occur in a minority of patients. In this report we first describe the rationale of the PROSPER/PHASE project and second show that the PROSPER/PHASE study can be used to study pharmacogenetics in the elderly.

**Methods:**

The genome wide association study (GWAS) was conducted using the Illumina 660K-Quad beadchips following manufacturer's instructions. After a stringent quality control 557,192 SNPs in 5,244 subjects were available for analysis. To maximize the availability of genetic data and coverage of the genome, imputation up to 2.5 million autosomal CEPH HapMap SNPs was performed with MACH imputation software. The GWAS for LDL-cholesterol is assessed with an additive linear regression model in PROBABEL software, adjusted for age, sex, and country of origin to account for population stratification.

**Results:**

Forty-two SNPs reached the GWAS significant threshold of p = 5.0e-08 in 5 genomic loci (APOE/APOC1; LDLR; FADS2/FEN1; HMGCR; PSRC1/CELSR5). The top SNP (rs445925, chromosome 19) with a p-value of p = 2.8e-30 is located within the APOC1 gene and near the APOE gene. The second top SNP (rs6511720, chromosome 19) with a p-value of p = 5.22e-15 is located within the LDLR gene. All 5 genomic loci were previously associated with LDL-cholesterol levels, no novel loci were identified. Replication in WOSCOPS and CARE confirmed our results.

**Conclusion:**

With the GWAS in the PROSPER/PHASE study we confirm the previously found genetic associations with LDL-cholesterol levels. With this proof-of-principle study we show that the PROSPER/PHASE study can be used to investigate genetic associations in a similar way to population based studies. The next step of the PROSPER/PHASE study is to identify the genetic variation responsible for the variation in LDL-cholesterol lowering in response to statin treatment in collaboration with other large trials.

## Background

Cardiovascular disease is the leading cause of death in industrialized countries at old age. Advancing age is one of the most important risk factors for cardiovascular disease [1]. With the rising number of elderly people in our society cardiovascular disease has a major impact on healthcare [2]. The prevention of cardiovascular disease is critically dependent on lipid lowering therapy including the 3-hydroxymethyl-3-methylglutaryl coenzyme A (HMG-CoA) reductase inhibitors (statins). Statins are the most prescribed class of drugs worldwide and therapy is generally associated with a reduction of cardiovascular events by 20-30%. However, clinical response is highly variable and adverse effects occur in a minority of patients [3]. Recent research provides evidence that genetic variation contributes importantly to this variable drug response [4].

Pharmacogenomics focuses on unraveling the genetic determinants of such variable drug responses, both in intended, beneficial effects and unintended, adverse effects [5]. Therefore, we here present the PHArmacogenetic study of Statin in the Elderly at risk (PHASE) a genome wide association study (GWAS) in the PROspective Study of Pravastatin in the Elderly at Risk for vascular disease (PROSPER)[6] investigating the genetic variation responsible for the individual variation in drug response funded by the European Union's Seventh Framework Programme. To validate the GWAS performed in the PHASE study, we executed a proof-of-principle study to investigate the underlying genetic variation in LDL cholesterol levels.

Recent GWA studies have identified several new loci that influence circulating levels of blood lipids with around 95 loci showing statistical associations with circulating total cholesterol levels, HDL cholesterol, LDL cholesterol, and triglycerides [7]. These GWA studies are executed in population based studies with various age groups, however the elderly (age > 75 years) are rarely represented in these studies. With this proof-of-principle study we provide a testing frame to show that the PROSPER/PHASE study has sufficient statistical power to find genome wide statistical significant associations in quantitative traits such as LDL cholesterol in an elderly population. We replicated our findings from the PROSPER/PHASE study in two independent cohorts to validate that our results contain no false positive findings.

## Methods

### Study population

PROSPER was an investigator-driven, prospective multi-national randomized placebo-controlled trial to assess whether treatment with pravastatin diminishes the risk of major vascular events in the elderly [6;8]. Between December 1997 and May 1999, we screened and enrolled subjects in Scotland, Ireland, and the Netherlands. Men and women aged 70-82 years were recruited if they had pre-existing vascular disease or were at increased risk of such disease because of smoking, hypertension, or diabetes. A total number of 5804 subjects, of whom more than 50% was female, were randomly assigned to pravastatin or placebo. Various clinical laboratory measurements were carried out like inflammatory markers (CRP and various cytokines) and other biochemical substrates (e.g. glucose, leptin) at baseline and during follow-up. The protocol of the PROSPER study meets the criteria of the Declaration of Helsinki and was approved by the Medical Ethics Committees of each participating institution. Written informed consent was obtained from all participating subjects.

### LDL cholesterol

Plasma lipids and lipoproteins were measured twice during the screening phase, i.e. at the beginning and end of the single-blind, placebo "run-in" phase according to the standardized Lipid Research Clinics protocol. Baseline LDL cholesterol levels were taken as the average of these 2 determinations prior to randomization to statin treatment. Total cholesterol (TC), HDL cholesterol, and triglycerides were assessed after an overnight fast, LDL cholesterol was calculated by the Friedewald formula, as previously described [8].

### Genotyping

**Figure 1:**
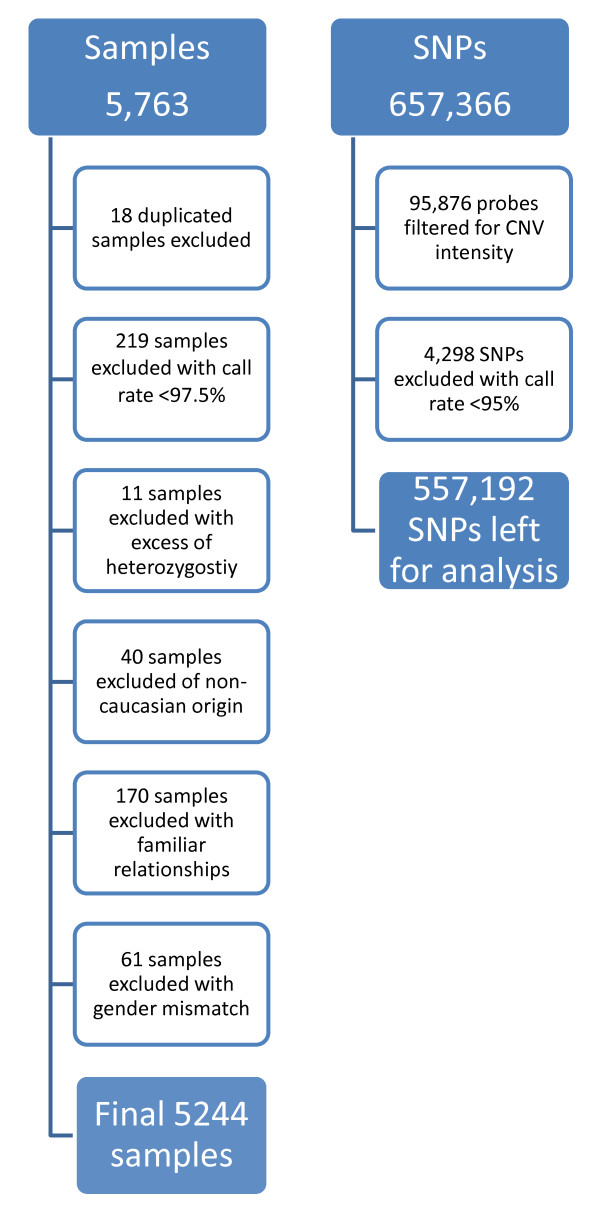
**Flow chart of the Quality Control of the PROSPER/PHASE study**.

The genotyping was conducted using the Illumina 660-Quad beadchips following manufacturer's instructions. These beadchips contain 657,366 single nucleotide polymorphism (SNP) and copy number variants (CNV) probes. After genotyping, samples and genetic markers were subjected to a stringent quality control protocol. From the 5763 samples with DNA available that underwent genotyping, 519 samples (9%) were excluded during the quality control (Figure 1). Excluded were 18 duplicated samples, 219 samples with a call rate < 97.5%, 11 samples with an excess for heterozygosity, 40 samples of non-caucasian origin, 170 samples with familiar relationships (IBD > 0.35), and 61 samples with a gender mismatch. From the 657,366 probes on the beadchips, 95,876 probes were filtered based on CNV intensity. Moreover, 4,298 SNPs were excluded with a call rate < 95%, leaving us with 557,192 SNPs for analysis. To maximize the availability of genetic data and coverage of the genome, imputation up to 2.5 million autosomal CEPH HapMap SNPs was performed with MACH imputation software based on the Hapmap built II release 23. To assess accuracy of the imputed genotypes, we compared the imputation output with SNPs that had been previously genotyped on other platforms.

### Statistical Analysis

Genome wide association analysis was performed with PROBABEL software specialized in genetic association analysis with imputed data taking the probability of the genotype into account (http://www.genabel.org/). With analyzing imputed genotypes, the observed allele count is replaced by the imputation's estimated dosage. For the continuous trait, baseline LDL cholesterol levels, an additive linear regression model was used to assess estimates and standard errors. The model was adjusted for sex and age, and country to correct for the within-study population structure. Standard errors for the regression estimates were calculated with model-robust methods. The analysis of 2.5 million SNPs at once poses a multiple testing problem. After the use of a Bonferroni correction, the threshold for genome wide significant results was set at 5.0e-08.

### Replication

Associations with a genome-wide significant p-value of 5.0e-08 were replicated in two independent cohorts, the West of Scotland Coronary Prevention Study (WOSCOPS)[9] and the Cholesterol and Recurrent Events (CARE) trial [10]. The WOSCOPS study was a double blind randomized placebo-controlled clinical trial in which 6595 men (age range 45-64 years)with hypercholesterolemia and no history of myocardial infarction were treated with 40 mg pravastatin (N = 3302) or placebo (N = 3293). GWAS data and baseline LDL cholesterol levels were available for 431 subjects. The CARE study was a double blind randomized placebo-controlled clinical trial in which 4159 patients (age range 21-75 years) were treated with 40 mg pravastatin (N = 2081) or placebo (N = 2078). GWAS data and baseline LDL cholesterol levels were available for 751 subjects. The significance level for the replication SNPs was set at p-value < 0.05.

## Results

Table 1 shows the baseline characteristics of the subjects participating in the PROSPER and the PROSPER/PHASE study. This table shows that the genotyped subjects in the PROSPER/PHASE study are representative of the total study population of the PROSPER study, since no major discrepancies exist between the two study sets. The mean age of all subjects at study entry was 75.3 years and about 50% of the participants were female.

**Table 1:** Baseline characteristics of the PROSPER/PHASE study

	PROSPER study (n = 5804)	PROSPER/PHASE study (n = 5244)
**Continuous variables (mean, SD)**		
Age (years)	75.3 (3.3)	75.3 (3.4)
Education (years)	15.1 (2.0)	15.1 (2.0)
Systolic blood pressure (mmHg)	154.7 (21.8)	154.6 (21.9)
Diastolic blood pressure (mmHg)	83.8 (11.5)	83.7 (11.4)
Height (cm)	165.2 (9.4)	165.2 (9.4)
Weight (kg)	73.4 (13.4)	73.3 (13.4)
Body mass index (kg/m^2)^	26.8 (4.2)	26.8 (4.2)
Total cholesterol (mmol/L)	5.7 (0.9)	5.7 (0.9)
LDL cholesterol (mmol/L)	3.8 (0.8)	3.8 (0.8)
HDL cholesterol (mmol/L)	1.3 (0.3)	1.3 (0.4)
Triglycerides (mmol/L)	1.5 (0.7)	1.5 (0.7)

**Categorical variables (n, %)**		
Males	2804 (48.3)	2524 (48.1)
Current smoker	1558 (26.8)	1392 (26.5)
History of diabetes	623 (10.7)	544 (10.4)
History of hypertension	3592 (61.9)	3257 (62.1)
History of angina	1559 (26.9)	1424 (27.2)
History of claudication	390 (6.7)	354 (6.8)
History of myocardial infarction	776 (13.4)	708 (13.5)
History of stroke or TIA	649 (11.2)	586 (11.2)
History of vascular disease*	2565 (44.2)	2336 (44.5)

*Any of stable angina, intermittent claudication, stroke, transient ischemic attack, myocardial infarction, peripheral artery disease surgery, or amputation for vascular disease more than 6 months before study entry.

**Figure 2:**
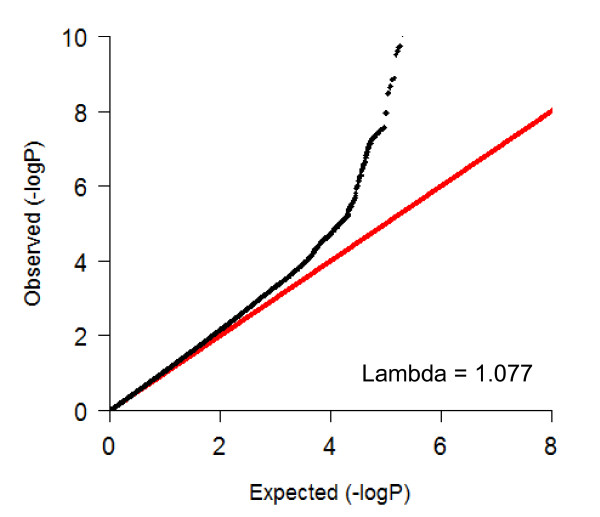
**QQ-plot for the GWAS on baseline LDL cholesterol in the PROSPER/PHASE study**.

**Figure 3:**
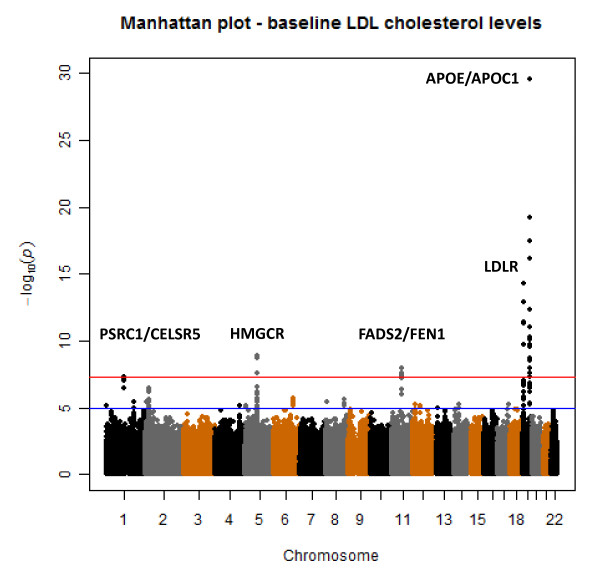
**Manhattan plot for the GWAS on baseline LDL cholesterol in the PROSPER/PHASE study**.

In Figure 2 the QQ-plot of the genome-wide association study with baseline LDL levels within the PROSPER/PHASE study is shown. In this plot it is shown that no genomic inflation has occurred in this analyses (lambda = 1.077) and that population stratification is sufficiently controlled for. In Figure 3 the results of the genome-wide association study with baseline LDL cholesterol levels within the PROSPER/PHASE study are depicted in a Manhattan plot. Forty-two SNPs in five genomic loci, APOE/APOC1, LDLR, FADS2/FEN1, HMGCR, and PSRC1/CELSR5, reached the genome-wide significant p-value of 5.0e-08. In table 2 a summary of the five genomic loci and their corresponding SNPs is given. The top SNP (rs445925, Chr. 19) with a p-value of p = 2.8e-30 is located within the APOC1 gene and near the APOE gene. Sixteen other SNPs in the same genomic region were also found to be associated with LDL cholesterol levels. The second top SNP (rs6511720, Chr. 19) with a p-value of p = 5.22e-15 is located within the LDLR gene. The three other genomic regions included the HMGCR (Chr.5), FADS2/FEN1 (Chr. 11), PSRC1/CELSR5 (Chr. 1) genes. All 5 genomic loci were previously found in association with LDL cholesterol levels and no novel loci were identified.

**Table 2:** Genomic loci with a genome wide significant p-value < = 5

**Chr**.	Gene	Number of SNPs	TopSNP	Variant	MAF	Beta	SE	p-value	Ref*
19	APOE APOC1	17	rs445925	G > A	0.11	-0.33	0.03	2.8e-30	(7;11-14;18;19)]
19	LDLR	5	rs6511720	G > T	0.13	-0.19	0.02	5.2e-15	(7;11;13;14;19)]
5	HMGCR	5	rs258494	G > C	0.38	0.10	0.02	1.3e-09	(7;11;13;14;19)]
11	FADS2 FEN1	14	rs174541	C > T	0.38	-0.10	0.02	1.1e-08	(7;11;13;19)]
1	PSRC1 CELSR5	1	rs602633	G > T	0.23	-0.11	0.02	5.0e-08	(7;11-14;16-19)]

Abbreviations: SNP, Single Nucleotide Polymorphism; Chr, Chromosome; MAF, minor allele frequency; SE, standard error. * A list of references in which the same loci were found.

We replicated the positive associations with genome-wide significant p-values in two independent cohorts, the WOSCOPS study and the CARE trial (table 3). Of our five genomic loci that were significantly associated with baseline LDL cholesterol levels we selected the top SNP for replication in both replication cohorts. If the SNP was not genotyped in their GWAS analysis, we chose a proxy in high linkage disequilibrium (r2 > 0.5%) for that SNP. These SNPs were associated with baseline LDL levels before randomisation to statin treatment in both studies. Three out of the five loci (APOE/APOC1; HMGCR; PSRC1/CELSR5) replicated in one or two replication cohorts (p < 0.05). The two other loci (LDLR and FADS2/FEN1) showed similar trends as shown in the discovery cohort, although they did not reach statistical significance (table 3).

**Table 3:** Replication of the 5 significant loci in the WOSCOPS trial and CARE study in association with baseline LDL cholesterol levels

			WOSCOPS N = 431			CARE N = 751		
			
SNP	Gene	**Chr**.	beta	se	p-value	beta	se	p-value
rs445925	APOE APOC1	19	0.07	0.05	0.164	-0.10	0.04	0.006
rs6511720	LDLR	19	-0.03	0.05	0.657	-0.03	0.03	0.411
rs258494*^1^	HMGCR	5	0.06	0.03	0.044	0.03	0.02	0.147
rs174541*^2^	FADS2 FEN1	11	-0.04	0.03	0.264	-0.03	0.02	0.134
rs602633*^3^	PSRC1 CELSR5	1	-0.09	0.04	0.026	-0.05	0.02	0.035

* A proxy for this SNP was used in both replication cohorts, for ^1 ^the proxy SNP was rs7715806 with a r^2 ^of 0.93, for ^2 ^the proxy SNP was rs174545 with a r^2 ^of 0.90, and for ^3 ^the proxy SNP was rs660240 with a r^2 ^of 0.88.

Abbreviations: SNP, Single Nucleotide Polymorphism; Chr, Chromosome.

## Discussion

With this first proof-of principle study we show that the PROSPER/PHASE GWAS can confirm previously found genetic associations with LDL-cholesterol levels. This proof-of-principle study indicates that the PROSPER/PHASE study is likely to be capable of detecting genomic regions responsible for the variation in various other quantitative traits. With almost 6000 samples in the PROSPER/PHASE study and access to various replication studies, the PROSPER/PHASE study can provide a good testing frame to identify the genetic variation responsible for the variation in LDL-cholesterol lowering in response to statin treatment.

The main locus responsible for the person-to-person variation in LDL-cholesterol levels is the chromosome 19 locus, which contains the APOE, APOC1, and LDLR genes. Other important loci included the HMGCR locus on chromosome 5, FADS2/FEN1 locus on chromosome 11, and the PSRC1/CELSR5 locus on chromosome 1. The five genomic loci that were associated with variation in LDL-cholesterol levels in the PHASE GWAS study were all genomic regions that were previously reported with LDL cholesterol variation [7;11-19]. Three out of the five loci were replicated in the WOSCOPS study and the CARE trial. The LDLR and FADS2/FEN1 loci were not replicated, however these loci were repeatedly found to be associated with LDL cholesterol levels in various other studies with large number of participants [7;11-14;16;19]. Moreover, both the WOSCOPS and CARE studies had genotype data available in a small number of subjects. Therefore, the lack of replication of these loci in WOSCOPS and CARE was most likely due to lack of statistical power. Finally, since we used in the replication studies a proxy SNP for some of the topSNPs, this may have diluted the effect.

## Conclusions

With this proof-of-principle study we show that the PROSPER/PHASE study can be used to investigate genetic associations in a similar way to population based studies. Moreover, we can also assume from these results that the PROSPER/PHASE study is likely to have sufficient power to detect genome-wide significant hits with large effects for other quantitative traits. The next step of the PROSPER/PHASE study is to identify the genetic variation responsible for the variation in LDL-cholesterol lowering in response to statin treatment.

## Competing interests

The authors declare that they have no competing interests.

## Authors' contributions

ST performed statistical analysis, interpretation of data, and drafted the manuscript. AdC performed statistical analysis, interpretation of data, and drafted the manuscript. IP performed statistical analysis and drafted the manuscript. IF, NS, DS, BB, JD, FS participated in design of the study and collected the data. MC carried out genotyping analyses. PS supervised the laboratory analysis and manuscript editing. RW participated in the design of the study and manuscript editing. JWJ participated in the design of the study, interpretation of the data, and manuscript editing. All authors read and approved the final manuscript.

## Pre-publication history

The pre-publication history for this paper can be accessed here:

http://www.biomedcentral.com/1471-2350/12/131/prepub

## References

[B1] McGovern PG Pankow JS Shahar E Doliszny KM Folsom AR Blackburn H Luepker RV Recent trends in acute coronary heart disease--mortality, morbidity, medical care, and risk factors. The Minnesota Heart Survey Investigators N Engl J Med 1996 334 14 884 90 10.1056/NEJM199604043341403 8596571

[B2] Kalantzi KJ Milionis HJ Mikhailidis DP Goudevenos JA Lipid lowering therapy in the elderly: is there a benefit? Curr Pharm Des 2006 12 30 3945 60 10.2174/138161206778559669 17073689

[B3] Kreisberg RA Oberman A Clinical review 141: lipids and atherosclerosis: lessons learned from randomized controlled trials of lipid lowering and other relevant studies J Clin Endocrinol Metab 2002 87 2 423 37 10.1210/jc.87.2.423 11836262

[B4] Johnson JA Cavallari LH Cardiovascular pharmacogenomics Exp Physiol 2005 90 3 283 9 10.1113/expphysiol.2004.028506 15778411

[B5] Goldstein DB Tate SK Sisodiya SM Pharmacogenetics goes genomic Nat Rev Genet 2003 4 12 937 47 14631354 10.1038/nrg1229

[B6] Shepherd J Blauw GJ Murphy MB Bollen EL Buckley BM Cobbe SM Ford I Gaw A Hyland M Jukema JW Kamper AM Macfarlane PW Meinders AE Norrie J Packard CJ Perry IJ Stott DJ Sweeney BJ Twomey C Westendorp RG Pravastatin in elderly individuals at risk of vascular disease (PROSPER): a randomised controlled trial Lancet 2002 360 9346 1623 30 10.1016/S0140-6736(02)11600-X 12457784

[B7] Teslovich TM Musunuru K Smith AV Edmondson AC Stylianou IM Koseki M Pirruccello JP Ripatti S Chasman DI Willer CJ Johansen CT Fouchier SW Isaacs A Peloso GM Barbalic M Ricketts SL Bis JC Aulchenko YS Thorleifsson G Feitosa MF Chambers J Orho-Melander M Melander O Johnson T Li X Guo X Li M Shin Cho Y Jin Go M Jin Kim Y Biological, clinical and population relevance of 95 loci for blood lipids Nature 2010 466 7307 707 13 10.1038/nature09270 20686565 PMC3039276

[B8] Shepherd J Blauw GJ Murphy MB Cobbe SM Bollen EL Buckley BM Ford I Jukema JW Hyland M Gaw A Lagaay AM Perry IJ Macfarlane PW Meinders AE Sweeney BJ Packard CJ Westendorp RG Twomey C Stott DJ The design of a prospective study of Pravastatin in the Elderly at Risk (PROSPER). PROSPER Study Group. PROspective Study of Pravastatin in the Elderly at Risk Am J Cardiol 1999 84 10 1192 7 10.1016/S0002-9149(99)00533-0 10569329

[B9] Shepherd J Cobbe SM Ford I Isles CG Lorimer AR Macfarlane PW McKillop JH Packard CJ Prevention of coronary heart disease with pravastatin in men with hypercholesterolemia. West of Scotland Coronary Prevention Study Group N Engl J Med 1995 333 20 1301 7 10.1056/NEJM199511163332001 7566020

[B10] Sacks FM Pfeffer MA Moye LA Rouleau JL Rutherford JD Cole TG Brown L Warnica JW Arnold JM Wun CC Davis BR Braunwald E The effect of pravastatin on coronary events after myocardial infarction in patients with average cholesterol levels. Cholesterol and Recurrent Events Trial investigators N Engl J Med 1996 335 14 1001 9 10.1056/NEJM199610033351401 8801446

[B11] Aulchenko YS Ripatti S Lindqvist I Boomsma D Heid IM Pramstaller PP Penninx BW Janssens AC Wilson JF Spector T Martin NG Pedersen NL Kyvik KO Kaprio J Hofman A Freimer NB Jarvelin MR Gyllensten U Campbell H Rudan I Johansson A Marroni F Hayward C Vitart V Jonasson I Pattaro C Wright A Hastie N Pichler I Hicks AA Loci influencing lipid levels and coronary heart disease risk in 16 European population cohorts Nat Genet 2009 41 1 47 55 10.1038/ng.269 19060911 PMC2687074

[B12] Barber MJ Mangravite LM Hyde CL Chasman DI Smith JD McCarty CA Li X Wilke RA Rieder MJ Williams PT Ridker PM Chatterjee A Rotter JI Nickerson DA Stephens M Krauss RM Genome-wide association of lipid-lowering response to statins in combined study populations PLoS One 2010 5 3 e9763. 20339536 10.1371/journal.pone.0009763 PMC2842298

[B13] Kathiresan S Melander O Guiducci C Surti A Burtt NP Rieder MJ Cooper GM Roos C Voight BF Havulinna AS Wahlstrand B Hedner T Corella D Tai ES Ordovas JM Berglund G Vartiainen E Jousilahti P Hedblad B Taskinen MR Newton-Cheh C Salomaa V Peltonen L Groop L Altshuler DM Orho-Melander M Six new loci associated with blood low-density lipoprotein cholesterol, high-density lipoprotein cholesterol or triglycerides in humans Nat Genet 2008 40 2 189 97 10.1038/ng.75 18193044 PMC2682493

[B14] Kathiresan S Willer CJ Peloso GM Demissie S Musunuru K Schadt EE Kaplan L Bennett D Li Y Tanaka T Voight BF Bonnycastle LL Jackson AU Crawford G Surti A Guiducci C Burtt NP Parish S Clarke R Zelenika D Kubalanza KA Morken MA Scott LJ Stringham HM Galan P Swift AJ Kuusisto J Bergman RN Sundvall J Laakso M Common variants at 30 loci contribute to polygenic dyslipidemia Nat Genet 2009 41 1 56 65 10.1038/ng.291 19060906 PMC2881676

[B15] Ma L Yang J Runesha HB Tanaka T Ferrucci L Bandinelli S Da Y Genome-wide association analysis of total cholesterol and high-density lipoprotein cholesterol levels using the Framingham heart study data BMC Med Genet 2010 11 55 20370913 10.1186/1471-2350-11-55 PMC2867786

[B16] Sandhu MS Waterworth DM Debenham SL Wheeler E Papadakis K Zhao JH Song K Yuan X Johnson T Ashford S Inouye M Luben R Sims M Hadley D McArdle W Barter P Kesäniemi YA Mahley RW McPherson R Grundy SM Wellcome Trust Case Control Consortium Bingham SA Khaw KT Loos RJ Waeber G Barroso I Strachan DP Deloukas P Vollenweider P Wareham NJ Mooser V LDL-cholesterol concentrations: a genome-wide association study Lancet 2008 371 9611 483 91 10.1016/S0140-6736(08)60208-1 18262040 PMC2292820

[B17] Wallace C Newhouse SJ Braund P Zhang F Tobin M Falchi M Ahmadi K Dobson RJ Marçano AC Hajat C Burton P Deloukas P Brown M Connell JM Dominiczak A Lathrop GM Webster J Farrall M Spector T Samani NJ Caulfield MJ Munroe PB Genome-wide association study identifies genes for biomarkers of cardiovascular disease: serum urate and dyslipidemia Am J Hum Genet 2008 82 1 139 49 10.1016/j.ajhg.2007.11.001 18179892 PMC2253977

[B18] Waterworth DM Ricketts SL Song K Chen L Zhao JH Ripatti S Aulchenko YS Zhang W Yuan X Lim N Luan J Ashford S Wheeler E Young EH Hadley D Thompson JR Braund PS Johnson T Struchalin M Surakka I Luben R Khaw KT Rodwell SA Loos RJ Boekholdt SM Inouye M Deloukas P Elliott P Schlessinger D Sanna S Genetic variants influencing circulating lipid levels and risk of coronary artery disease Arterioscler Thromb Vasc Biol 2010 30 11 2264 76 10.1161/ATVBAHA.109.201020 20864672 PMC3891568

[B19] Willer CJ Sanna S Jackson AU Scuteri A Bonnycastle LL Clarke R Heath SC Timpson NJ Najjar SS Stringham HM Strait J Duren WL Maschio A Busonero F Mulas A Albai G Swift AJ Morken MA Narisu N Bennett D Parish S Shen H Galan P Meneton P Hercberg S Zelenika D Chen WM Li Y Scott LJ Scheet PA Newly identified loci that influence lipid concentrations and risk of coronary artery disease Nat Genet 2008 40 2 161 9 10.1038/ng.76 18193043 PMC5206900

